# Median 10 years follow-up of patients with covert Cushing’s syndrome: a case series

**DOI:** 10.1186/s13256-021-03046-3

**Published:** 2021-10-11

**Authors:** Farzad Najafipour, Amir Bahrami, Mitra Niafar, Jalil Houshyar, Monireh Halimi, Vahideh Sadra

**Affiliations:** 1 Endocrine Research Center, Tabriz University of Medical Sciences, Tabriz, Iran; 2 Department of Clinical Pathology, Faculty of Medicine, Tabriz University of Medical Sciences, Tabriz, Iran; 3 Department of Internal Medicine, Faculty of Medicine, Tabriz University of Medical Sciences, Tabriz, Iran

**Keywords:** Case report, Ectopic ACTH secretion syndrome, Cushing’s syndrome

## Abstract

**Background:**

Ectopic adrenocorticotropic hormone secretion syndrome occurs in 10% of all patients with adrenocorticotropic-hormone-dependent hypercortisolism. It is usually associated with overt malignancies or with occult and indolent tumors. This study aims to confirm the source of ectopic adrenocorticotropic hormone in four patients with ectopic Cushing’s syndrome over time.

**Case presentation:**

A 38-year-old Iranian man with Cushing’s syndrome underwent bilateral adrenalectomy since the source of ectopic adrenocorticotropic hormone secretion was not localized and pituitary imaging was normal. A whole-body scan revealed a right-lung tumoral mass with mediastinal lymph node metastasis. The mass was assumed a lung carcinoid tumor with mediastinal adenopathy. Right-lung mid-zone lobectomy and mediastinal lymphadenectomy were done. In a 47-year-old Iranian man with Cushing’s syndrome, whole-body computed tomography scan revealed a pulmonary nodule in the posterior segment of the left lower lobe of the lung. The third case was a 25-year-old Iranian man who presented with symptoms and signs of Cushing’s syndrome. Pituitary magnetic resonance imaging revealed a microadenoma 5 × 9 mm. Whole-body scan showed abnormal focal somatostatin receptors analog avid lesion in the posterior aspect of inferior third of right lung, highly suggestive of ectopic adrenocorticotropic-hormone-producing tumor. The last case was a 43-year-old Iranian woman with Marfan syndrome with a history of mitral and aortic valve replacement and chronic dissection of the aorta, who presented with symptoms and signs of Cushing’s syndrome. She underwent bilateral adrenalectomy 1 year later owing to failure to locate ectopic adrenocorticotropic hormone syndrome. Whole-body scan showed abnormally increased radiotracer uptake in the midline of the skull base and posterior aspect of the middle zone of left hemithorax and bed of left lobe of thyroid.

**Conclusion:**

The clinical spectrum of ectopic adrenocorticotropic hormone secretion syndrome is wide, and distinguishing Cushing’s disease from ectopic adrenocorticotropic hormone secretion syndrome is difficult. Initial failure to identify a tumor is common. Pulmonary carcinoid or occult source of ectopic adrenocorticotropic hormone secretion syndrome is usually the cause. In occult cases of ectopic adrenocorticotropic hormone in which the tumor cannot be localized, serial follow-up with serial computed tomography, magnetic resonance imaging, or scintigraphy is recommended for several years until the tumor can be localized and treated.

## Introduction

Harvey Cushing described Cushing’s syndrome for the first time in 1912. Since then, diagnosis and management of Cushing’s syndrome has been a controversial topic [1]. Cushing’s syndrome is characterized by a group of symptoms and signs caused by chronic exposure to excess exogenous or endogenous glucocorticoids. Due to increased morbidity and mortality in Cushing’s syndrome, early diagnosis and treatment may prevent symptom progression, and reduce morbidity and mortality. The diagnosis is biochemical and based on screening and confirmatory laboratory tests [2].

The syndrome is categorized into adrenocorticotropic hormone (ACTH)-dependent and ACTH-independent forms. The most common cause of Cushing’s syndrome is exogenous Cushing’s syndrome, so, initially, exogenous Cushing’s should be excluded [3]. Cushing’s disease is the most common cause of ACTH-dependent Cushing’s syndrome. In 90% of cases, the cause of the disease is the pituitary secreting ACTH microadenoma [4, 5]. Ectopic ACTH secretion syndrome (EAS) occurs in 10% of all patients with ACTH-dependent hypercortisolism [6].

The classic features of Cushing’s syndrome include central obesity, proximal muscle weakness, moon face, facial plethora, easy bruising, hirsutism, purplish striae, and menstrual irregularity. Psychiatric disorders may be absent in ectopic ACTH syndrome [6, 7].

Rapid clinical evolution, dominantly proximal myopathy, slight central distribution of body fat, hypertension, peripheral edema, hypokalemia, hyperpigmentation, and glucose intolerance in patients are indicative of ectopic ACTH syndrome [8]. Hypokalemia occurs in 80% of described cases and is usually more severe than in Cushing’s disease [9, 10].

This study aimed to present our experience with EAS patients for a median of 10-year follow-up to assist physicians with the difficulties in diagnosis and management of these cases.

### Patient consent and ethical approve

The study protocol was approved by the ethics committee of Tabriz University of Medical Science, Tabriz, Iran. This research complied with the standards of the Declarations of Helsinki and current ethical guidelines. Informed consent has been obtained from the patients for publication of the case series and accompanying images and patient data.

### Case report 1

**Fig. 1:**
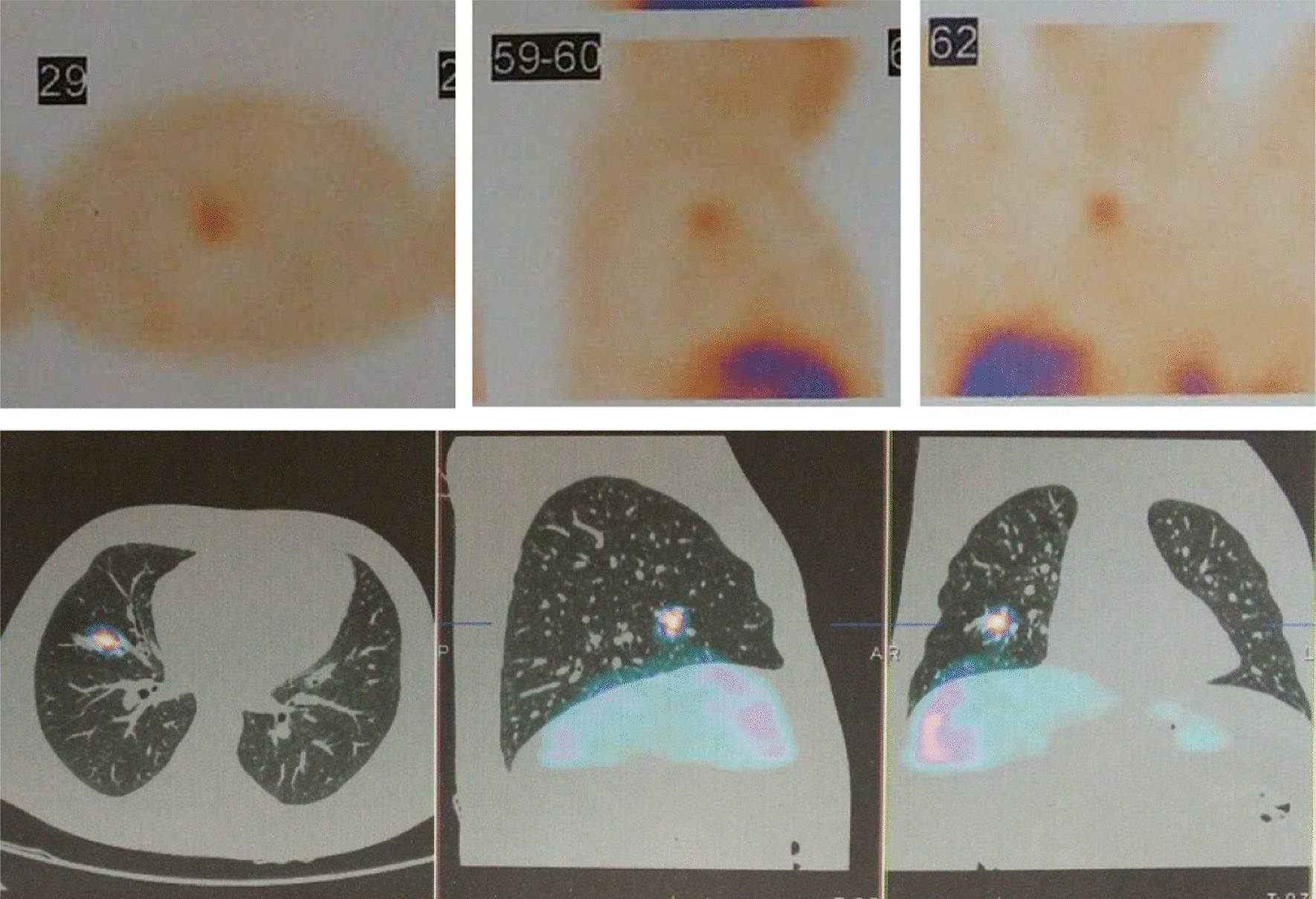
TC99-HYNIC-Toctrotide scintigraphy: right-lung tumoral mass with mediastinal lymph node metastasis

**Fig. 2:**
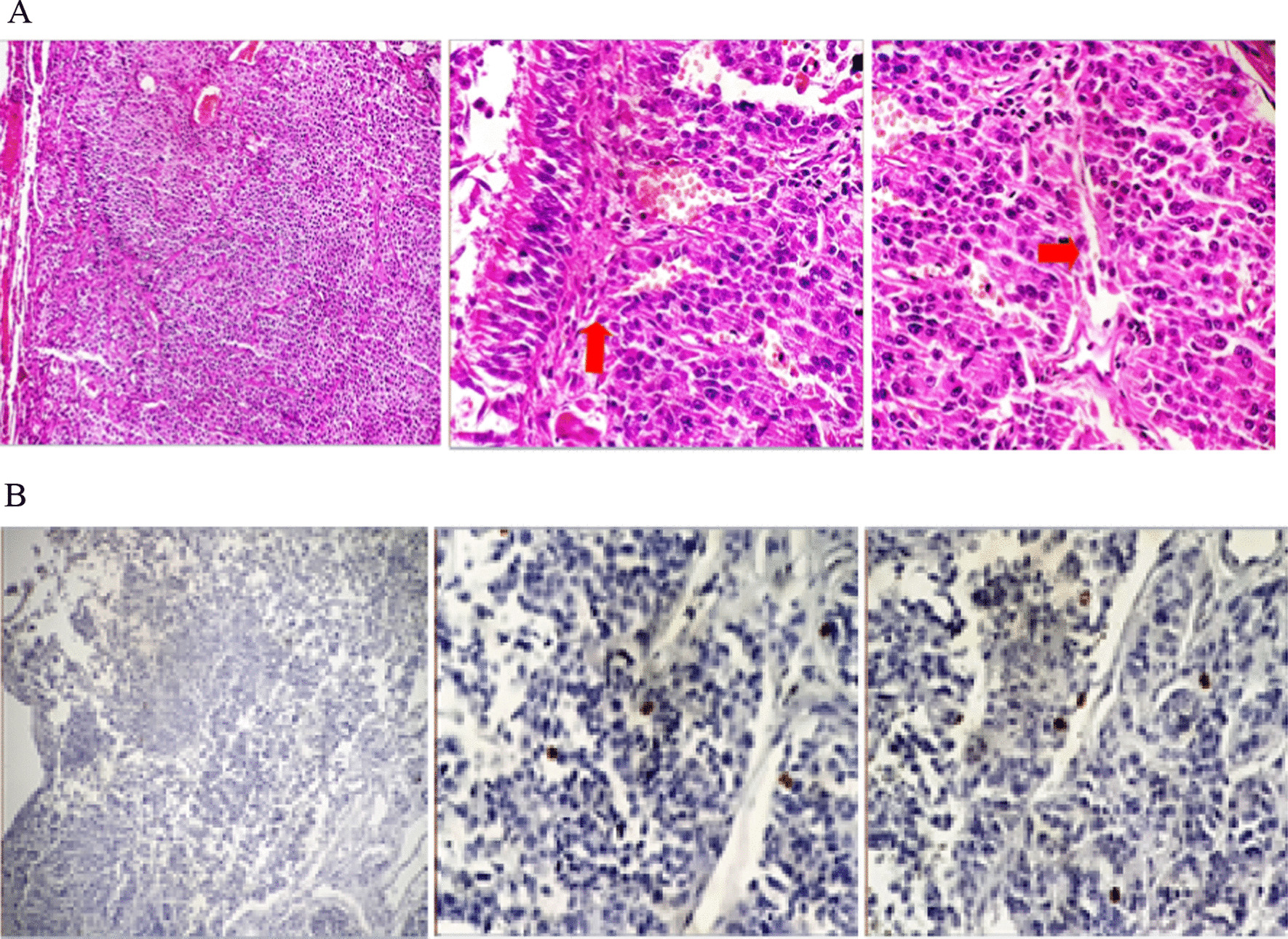
Pathological images of the tumor. **A** Hematoxylin and eosin (H&E) staining, uniform neoplastic cells, small to medium in size with scant cytoplasm, oval nuclei with coarse chromatin. Well-differentiated neuroendocrine tumor, grade 1; vascular invasion is seen. **B** IHC and Ki67 staining, positive in 2% tumoral cells

**Fig. 3:**
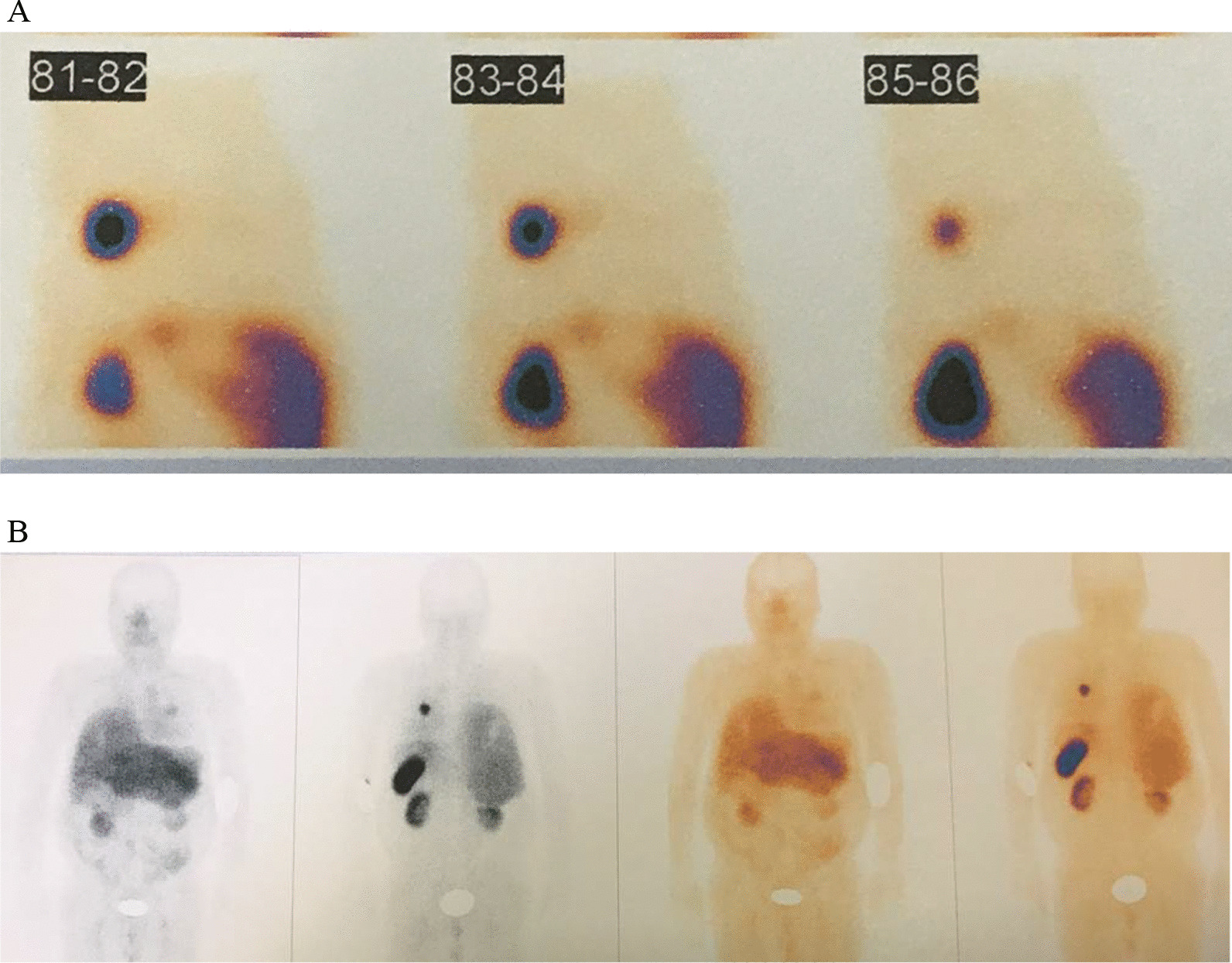
99-HYNIC-Toctrotide scintigraphy: abnormal increased radiotracer uptake in lower lobe of left lung **A**, **B**

A 38-year-old Iranian man with known case of Cushing’s syndrome (ACTH-dependent form) was clinically and biochemically confirmed 16 years ago. He underwent bilateral adrenalectomy 15 years ago since the source of ectopic ACTH secretion was not localized and pituitary imaging was normal. He received 5 mg of prednisolone and 0.1 mg fludrocortisone daily since then. After bilateral adrenalectomy, all symptoms and signs were improved except for hyperpigmentation. He was on regular follow-up and used his medications regularly. He presented with a 1-year history of diabetes, hypertension, weight gain of 5 kg, worsening of skin pigmentation, proximal myopathy, and new abdominal striae. Initially, exogenous Cushing’s syndrome was supposed, so the steroid dose was decreased to 2.5 mg/daily. Symptoms and signs of Cushing’s syndrome were not improved. Prednisolone was stopped, and new investigations were done for the patient after admission to the endocrine ward. His basal morning cortisol level was 37 μg/dl, his morning cortisol level after 1 mg overnight dexamethasone suppression test was 40 μg/dl, and his corticotropin level was 910 pg/ml. Twenty-four-hour urine free cortisol was 158 μg/24 hours, and 24-hour urine Cr was 900 mg/24 hours. Pituitary magnetic resonance imaging (MRI) was normal, and an ectopic source of ACTH was suspected. Axial computed tomographic images of the chest revealed a 9-mm paratracheal lymph node and a band-shaped consolidation in the right middle lobe. A 28-mm mass indicative of right adrenal hyperplasia was reported on abdominal computed tomography (CT) scan. A whole-body scan with TC99-HYNIC-Toctrotide revealed a right lung tumoral mass with mediastinal lymph node metastasis (Fig. 1). The mass was assumed to be a lung carcinoid tumor with mediastinal adenopathy. Right-lung mid-zone lobectomy and mediastinal lymphadenectomy were done. Histologic study of right-lung lobe mass revealed a well-differentiated neuroendocrine tumor, grade 1 with vascular invasion. Mitosis was 1/10 high-power field (HPF) (Fig. 2). In addition, Ki67 was positive in 2% of tumoral cells, and 13 out of 21 lymph nodes were affected by tumor (Fig. 3). These findings confirmed a diagnosis of corticotrophin-producing lung carcinoid tumor.

The postoperative level of serum cortisol was 0.16 μg/dl, and corticotrophin was 19.9 pg/ml. Three months after surgery, the patient had nearly complete clinical recovery. Table 1 presents the laboratory finding of case 1.

**Table 1:** Laboratory findings of case 1

Parameter	Level/concentration
Hb (mg/dl)	15.7
Hct (%)	45
MCV (femtoliter)	84
TSH (mIU/L)	0.5
FT4 (ng/dl)	1.5
Potassium (mEq/L)	4
Total calcium (mg/dl)	9
Creatinine (mg/dl)	0.8
Basal cortisol (8 AM) (µg/dl)	37
1 mg overnight dexamethasone suppression test (µg/dl)	40
ACTH (pg/ml)	910
UFC (µg/dl/24 hours)	158

*Hb* hemoglobin, *Hct* hematocrit, *MCV* mean cell volume, *TSH* thyroid stimulating hormone, *FT4* free T4, *ACTH* adrenocorticotropic hormone, *UFC* urine free cortisol

### Case report 2

A 47-year-old Iranian man with Cushing’s syndrome (ACTH-dependent) was clinically and biochemically confirmed 15 years ago. Because of normal pituitary imaging, and unavailability of facilities to perform IPSS, ectopic Cushing’s syndrome was suspected, and a whole-body CT scan revealed a pulmonary nodule in the posterior segment of the left lower lobe of the lung. Biopsy of pulmonary nodule was done, revealing a chronic inflammatory lesion.

Therefore, the patient underwent bilateral adrenalectomy 15 years ago. Serial chest CT scans showed no change in size and number of pulmonary nodules over time. The hyperpigmentation was worsened at the last follow-up visit, and ACTH levels were 620, 721, 910, and 1500 pg/ml, respectively.

**Fig. 4:**
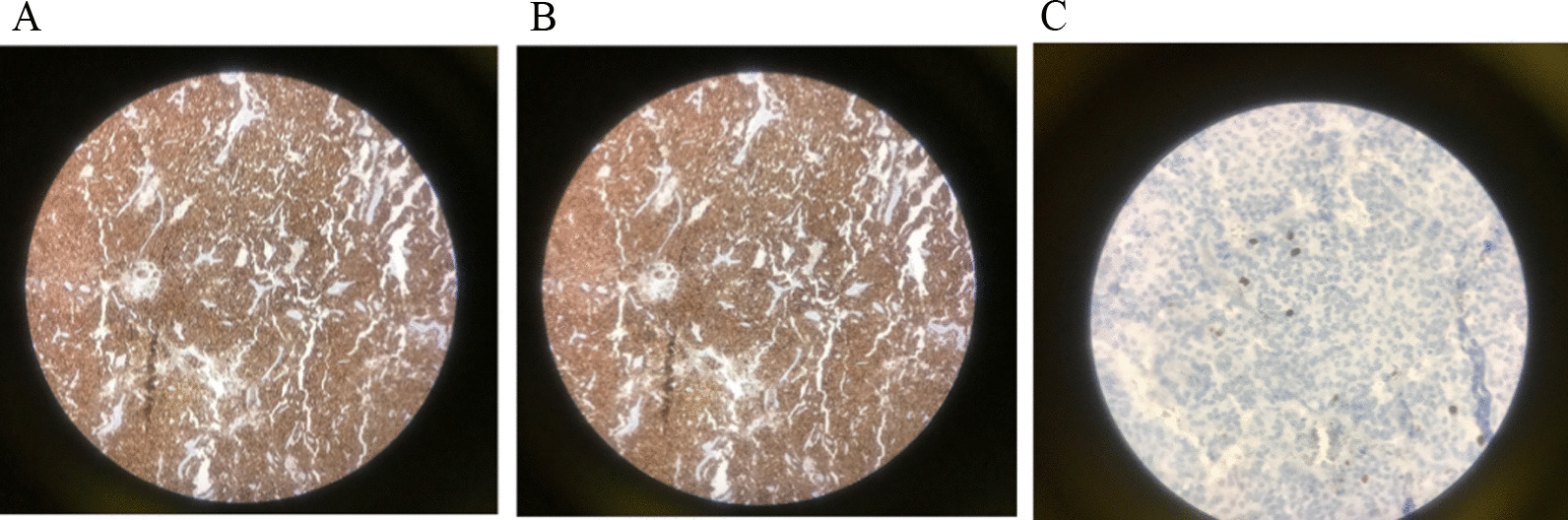
Pathological images of the tumor. **A** Microscopic picture H&E staining: uniform neoplastic cells, small to medium in size with scant cytoplasm, oval nuclei with coarse chromatin bland tumor cells in solid pattern indicative of low-grade neuroendocrine tumor. **B** Chromogranin staining. **C** Synaptophysin staining

Dynamic MRI of the pituitary gland with and without contrast was normal. Whole-body scan and single-photon emission computerized tomography (SPECT) with TC 99-HYNIC-Toctrotide showed abnormally increased radiotracer uptake in the lower lobe of the left lung (Fig 3). The last chest CT scan showed a hyperdense solid mass in the lower lobe of the left lung. The patient underwent thoracotomy, and left lower lobectomy was done. Histologic study of left lung mass revealed a low-grade neuroendocrine tumor with pleural invasion and without vascular invasion. Immunohistochemistry (IHC) revealed positive chromogranin, TTF1, and synaptophysin staining, and Ki67 was 1–2% positive in favor of low-grade neuroendocrine tumor (Fig 4). Table 2 presents the laboratory finding of case 2.

**Table 2:** Laborato﻿ry findings of case 2

Parameter	Concentration
Basal cortisol (8 AM) (µg/dl)	18.9
Cortisol after 1 mg overnight dexamethasone suppression test (µg/dl)	12.9
Cortisol after low-dose dexamethasone suppression test (µg/dl)	9.6
ACTH (1–4) (pg/ml)	620, 721, 910, and 1500

*ACTH* adrenocorticotropic hormone

### Case report 3

**Fig. 5:**
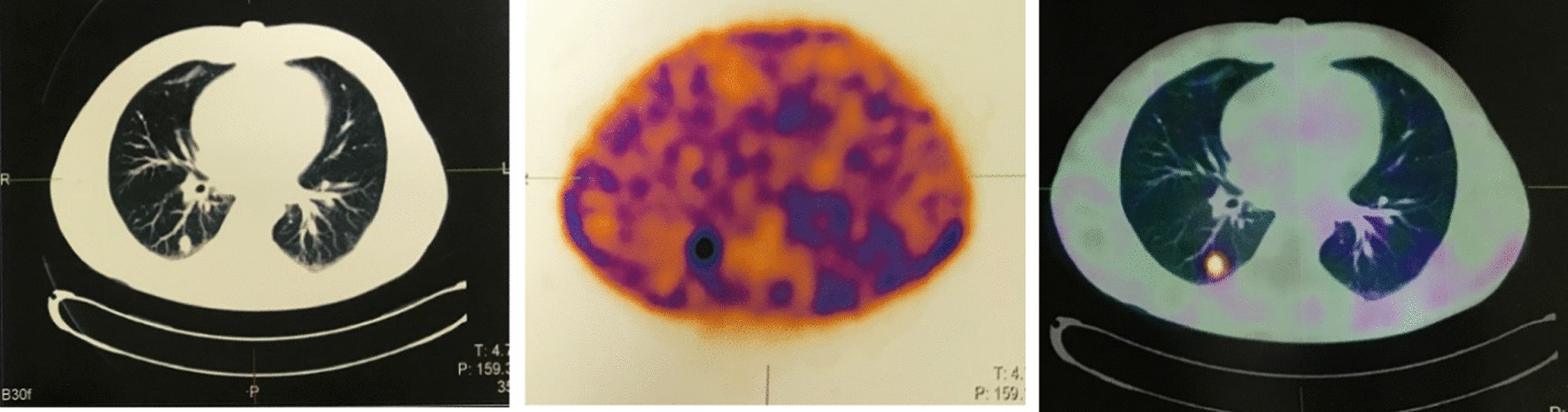
Tc99m Octreotide (HYNIC-Toc) scintigraphy: abnormal focal somatostatin receptors analogue avid lesion in posterior aspect of inferior third of right lung, highly suggestive of ectopic ACTH-producing tumor

A 25-year-old Iranian man presented with symptoms and signs of Cushing’s syndrome (weight gain, proximal muscle weakness, abdominal purplish striae, hypertension, diabetes mellitus, and fragility fractures in thoracic and lumbar spines due to osteoporosis; *Z* score-5.1 in the lumbar area). An endocrine evaluation showed corticotropin-dependent hypercortisolism (ACTH level was 250 pg/ml). Pituitary MRI revealed a microadenoma 5 × 9 mm. Because of the unavailability of facilities to perform inferior petrosal sinus sampling (IPSS), the patient underwent transsphenoidal surgery. Three months later, hypercortisolism persisted. An ectopic source could not be localized. The whole-body CT scan was unremarkable. Bilateral adrenalectomy was performed. Four year later, follow-up chest CT scan revealed a 17 × 11 mm mass in the posterior basal segment of the right lung. Whole-body scan and single-photon emission computerized tomography (SPECT) with Tc99m Octreotide (HYNIC-Toc) showed abnormal focal somatostatin receptors analog avid lesion in the posterior aspect of inferior third of right lung, highly suggestive of ectopic ACTH-producing tumor (Fig. 5). The patient underwent thoracotomy, and wedge resection of the right lower lobe was done. Histologic study of right lower lobe mass revealed neuroendocrine tumor that was well differentiated (consistent with carcinoid tumor), and IHC staining for VSE, Ki67, and chromogranin A was positive.

### Case report 4

A 43-year-old Iranian woman with Marfan syndrome with a history of mitral and aortic valve replacement and chronic dissection of the aorta presented with symptoms and signs of Cushing’s syndrome, including weight gain, proximal muscle weakness, abdominal purplish striae, easy disability, and osteoporosis 7 years ago.

ACTH-dependent hypercortisolism was confirmed. Dynamic MRI of the pituitary gland with and without contrast was normal. Chest, abdomen, and pelvic imaging were unremarkable.

She underwent bilateral adrenalectomy 1 year later owing to failure to locate ectopic ACTH syndrome. Patient follow-ups revealed worsening of hyperpigmentation and increased level of ACTH to 285 pg/ml.

**Fig. 6:**
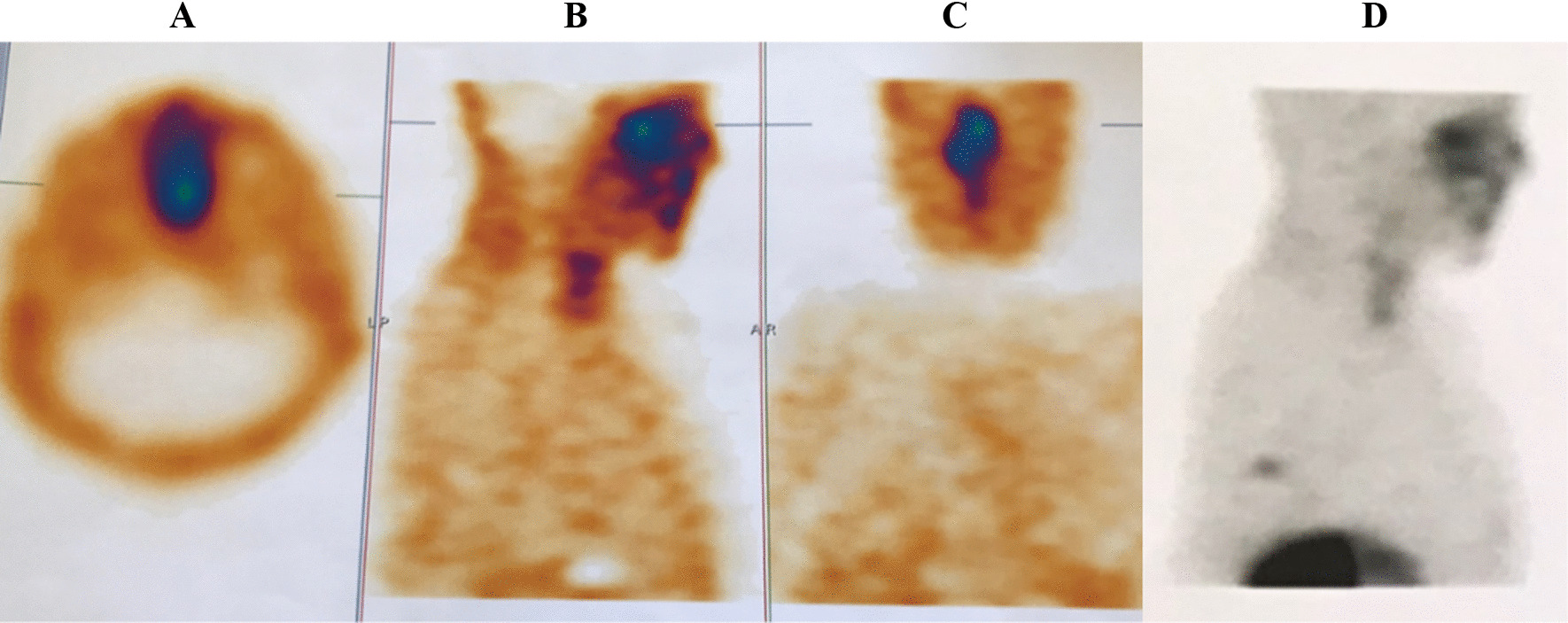
Whole body scan and SPECT with Tc99m–HYNIC-T Octreotide early and delayed images: abnormal increased radiotracer uptake in **A** midline of skull base, **B** bed of left lobe of thyroid, and **C**, **D** posterior aspect of middle zone of left hemithorax

Whole-body scan and SPECT with Tc99m–HYNIC-T Octreotide early and delayed images showed abnormally increased radiotracer uptake in the midline of the skull base and posterior aspect of the middle zone of left hemithorax and bed of left lobe of thyroid (Fig. 6). Ga-Dotatate PET/CT scan revealed somatostatin receptor-positive pulmonary lesion (likely neuroendocrine tumor) in the superior segment of the left lower lobe.

**Fig. 7:**
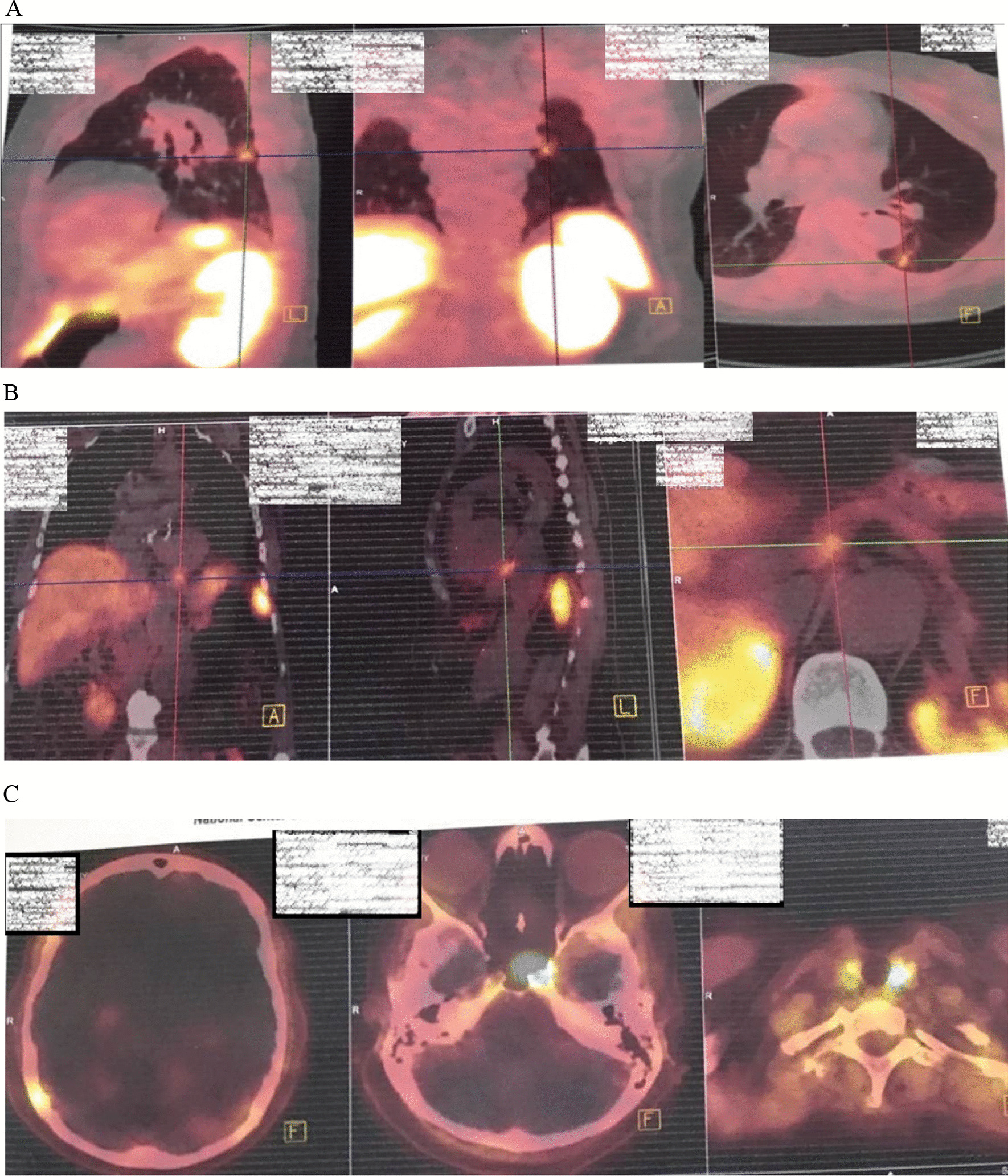
Ga-Dotatate PET/CT scan. **A** Somatostatin receptor positive pulmonary lesion (likely neuroendocrine tumor) in superior segment of the left lower lobe. **B** Two somatostatin receptor positive (SRP) tumoral lesions inferior to the gastroesophageal junction and in close contact with uncinate process of the pancreas. **C** Multiple somatostatin receptor positive (SRP) tumoral lesions in right parietal bone and dorsal frontal bones

Two somatostatin receptor-positive (SRP) tumoral lesions inferior to the gastroesophageal junction and in close contact with the uncinate process of the pancreas were detected (Fig. 7A). Multiple somatostatin receptor-positive (SRP) tumoral lesions in the right parietal bone and dorsal frontal bones were seen (Fig. 7B).The patient was treated with Sandostatin LAR as a suspected case of metastatic NET. Three months later, ACTH level decreased to 58 pg/ml (Fig. 7C).

## Discussion

Ectopic ACTH secretion, causing Cushing’s syndrome, accounts for 10–20% of ACTH-dependent Cushing’s cases [4]. Ectopic Cushing’s syndrome has been reported across a wide age range, including elderly and young people, and there is a wide range of tumors in terms of origin and size [7, 11]. The most commonly presented symptoms and signs are proximal myopathy in 95%, hyperpigmentation 90%, easy bruising 67%, edema 57%, and stria 52% of cases; hypokalemia and central obesity are uncommon.

Ectopic ACTH syndrome is classified into overt form, in which the source of ACTH is clear on diagnostic workups; covert form, in which the source is not apparent on initial investigation but is finally localized; and occult, in which source of ACTH could not be discovered [4]. The average time for localization of the source of ACTH in our study was about 10 years.

Multiple tumors, such as neuroendocrine tumors (NET), islet cell tumors, medullary thyroid cancer, small-cell lung cancer, pheochromocytoma, and thymic and pancreatic carcinoids, can secret ectopic ACTH and cause Cushing’s syndrome [8]. In 45% of cases, the origin of ectopic ACTH is in the chest, of which 25% account for bronchial carcinoid tumors [8]. The origin of ectopic ACTH was in the chest in all our cases.

Localization of carcinoid tumors is usually challenging because of their small size and relatively slow-growing nature. Conventional imaging studies such as CT and MRI can detect tumors in only 50% of cases. Nuclear medicine improves our diagnostic approach when the identification of tumors is not possible in conventional radiologic studies. In covert cases, nuclear imaging identifies 79.1% of tumors not seen on conventional imaging’s [12]. Sensitivity differs according to the tumor site and localization techniques [12].

The standard Octreotide scan has 49% sensitivity [11, 12]. In overt cases, CT has 98.3% sensitivity and fluorodeoxyglucose–positron emission tomography (FDG-PET) has 71% sensitivity [13]. However, in covert cases, gallium–somatostatin receptor PET/CT (SSTR-PET/CT) shows the greatest sensitivity of 100%, MRI 44.8%, and CT 43.3%. The sensitivity is different according to tumor site; for example, lung lesions are identified with the greatest sensitivity by CT (79%), MRI (66.7%), gallium–SSTR-PET/CT (77.8%) [12]. Thyme tumors are identified by optical coherence tomography (OCT) (85.7%), CT (85%), MRI (62.5%), and FDG-PET (62.5%). Pancreatic lesions are found by FDG-PET (100%), MRI (87.5%), and CT (86.7%). Gastrointestinal tract lesions are identified by CT (90%) and MRI (71.4%) [12, 14, 15].

In our study, the source of ectopic ACTH syndrome was confirmed by scintigraphy in all cases. NETs have specific immunohistochemistry features. Chromogranin A, Synaptophysin, and Ki67 are usually positive [8], and were positive in three cases of our study.

## Conclusions

We conclude that the clinical spectrum of EAS is wide. Sometimes distinguishing Cushing’s disease from EAS is difficult, and localization of ectopic ACTH source is challenging. Initial failure to identify a tumor is common. Pulmonary carcinoid or occult source of EAS is usually the cause. In occult cases of ectopic ACTH in which the tumor cannot be localized, serial follow-up with serial CT, MRI, or scintigraphy is recommended for several years until the tumor can be localized and treated.

## Data Availability

The datasets used and/or analyzed during the current study are available from the corresponding author on reasonable request

## Abbreviations

ACTH: Adrenocorticotropic hormone
EAS: Ectopic ACTH secretion syndrome
MRI: Magnetic resonance imaging
CT: Computed tomography
IHC: Immunohistochemistry
SPECT: Single-photon emission computerized tomography
IPSS: Inferior petrosal sinus sampling
SRP: Somatostatin receptor-positive
NET: Neuroendocrine tumors
FDG-PET: Fluorodeoxyglucose–positron emission tomography
SSTR-PET/CT: Somatostatin receptor PET/CT
OCT: Optical coherence tomography
